# Big Data, Small Stories: Methodological Considerations for Using Social Media Analytics in Medical Education Research

**DOI:** 10.2196/94825

**Published:** 2026-06-01

**Authors:** Xiaoshuai Li, Rui Wang

**Affiliations:** 1 Department of Blood Transfusion, Shengjing Hospital of China Medical University, Shenyang, Liaoning, China; 2 Department of Stem Cells and Regenerative Medicine, Basic Medical Sciences of China Medical University, NO.77 Puhe Road, Shenyang, Liaoning, 110122, China, 86 18900911141

**Keywords:** Icarus paradox, aspirations, realities, medical education, student well-being, community perspective, online reviews

I read with great interest the recent article by Binsar and Hamsal [1], who innovatively applied the Icarus paradox framework to examine Indonesia’s specialist medical education system through the lens of online public reviews. The use of social listening tools to capture 5047 reviews across multiple platforms represents a timely methodological contribution to the field of digital health professional education. I commend the authors for their ambitious data collection and for highlighting the critical tension between aspirations and reality during medical training.

While Binsar and Hamsal’s [1] study makes a valuable contribution by introducing the Icarus paradox framework to medical education research and leveraging large-scale social media data, its heavy reliance on automated sentiment analysis raises a fundamental methodological question that warrants deeper scrutiny. Neutral sentiment dominated the dataset, comprising 94.9% of news portal reviews, 93.2% of blog posts, and 86.6% of website mentions. While the authors interpret this as “public discourse on specialist medical education tends to be descriptive or informational rather than overtly evaluative,” this neutrality more likely reflects inherent limitations of automated analysis in capturing context-dependent meanings—a methodological blind spot that surface-level polarity detection cannot address. Previous research demonstrates that discussions about professional training often carry latent meanings—such as tacit acceptance of systemic pressures, veiled critique, or discursive normalization—that simple sentiment classification inevitably misses [2-4].

To move beyond this impasse, we propose a more rigorous and integrated methodological pathway. Future studies should use a sequential explanatory design that operates in two phases. In the first phase, social listening tools would be used to map the discursive landscape and identify sentiment polarities as well as “sentiment silence zones”—clusters of highly neutral discourse in which meaning may be latent rather than explicit. In the second phase, purposive sampling would be based on the findings of the first phase, selecting representative texts from these silence zones for in-depth qualitative analysis using methods such as critical discourse analysis or narrative inquiry. This “quantitative mapping, qualitative mining” strategy would enable researchers to ask not merely “What sentiments are being expressed?” but also “What cultural work is this discourse performing?” and “How might apparently neutral language tacitly reproduce or contest systemic tensions in medical education?”

Recent methodological innovations in health professions education [2-4] offer rigorous frameworks for accessing qualitative depth. Kahlke et al [2] demonstrate how innovative elicitation techniques can access tacit dimensions of professional experience, Kinnear et al [3] reveal how apparently neutral professional discourse conceals deep assumptions about educational quality, and MacLeod et al [4] provide systematic frameworks for understanding how language constitutes professional realities. Their deliberate integration with computational social listening represents the next frontier for the field.

Despite this methodological caveat, this study makes an important contribution by amplifying public voices. Understanding the Icarus paradox ultimately requires methods capable of capturing how systemic aspirations and vulnerabilities are lived and articulated in everyday discourse. We look forward to seeing this line of inquiry evolve.

## References

[1] Binsar F, Hamsal M (2026). Exploring the Icarus paradox in Indonesia’s specialist medical education system using the public perspective from online media: convergent mixed methods study. JMIR Med Educ.

[2] Kahlke R, Maggio LA, Lee MC (2025). When words fail us: an integrative review of innovative elicitation techniques for qualitative interviews. Med Educ.

[3] Kinnear B, Schumacher DJ, Varpio L, Driessen EW, Konopasky A (2024). Legitimation without argumentation: an empirical discourse analysis of “Validity as an Argument” in assessment. Perspect Med Educ.

[4] MacLeod A, Ellaway RH, Cleland J (2024). A meta-study analysing the discourses of discourse analysis in health professions education. Med Educ.

